# Prevalence of Chemotherapy-Induced Peripheral Neuropathy in Multiple Myeloma Patients and its Impact on Quality of Life: A Single Center Cross-Sectional Study

**DOI:** 10.3389/fphar.2021.637593

**Published:** 2021-04-22

**Authors:** Marie Selvy, Nicolas Kerckhove, Bruno Pereira, Fantine Barreau, Daniel Nguyen, Jérôme Busserolles, Fabrice Giraudet, Aurélie Cabrespine, Carine Chaleteix, Martin Soubrier, Jacques-Olivier Bay, Richard Lemal, David Balayssac

**Affiliations:** ^1^ INSERM U1107 NEURO-DOL, Université Clermont Auvergne, Clermont-Ferrand, France; ^2^ Service de Chirurgie digestive, CHU Clermont-Ferrand, Clermont-Ferrand, France; ^3^ Service de Pharmacologie, CHU Clermont-Ferrand, Clermont-Ferrand, France; ^4^ Institut Analgesia, Université Clermont Auvergne, Clermont-Ferrand, France; ^5^ CHU Clermont-Ferrand, Direction de La Recherche Clinique et de l’Innovation, Clermont-Ferrand, France; ^6^ Service d’Hématologie clinique adulte, CHU Clermont-Ferrand, Clermont-Ferrand, France; ^7^ College of Pharmacy, University of Oklahoma, Oklahoma City, OK, United States; ^8^ Service de Rhumatologie, CHU Clermont-Ferrand, Clermont-Ferrand, France; ^9^ UNH-UMR 1019, INRA, Université Clermont Auvergne, Clermont-Ferrand, France; ^10^ EA 7453 CHELTER, Université Clermont Auvergne, Clermont-Ferrand, France

**Keywords:** bortezomib, multiple myeloma, chemotherapy-induced peripheral neuropathy, neuropathic pain, health-related quality of life, anxiety, depression

## Abstract

Bortezomib is a pivotal drug for the management of multiple myeloma. However, bortezomib is a neurotoxic anticancer drug responsible for chemotherapy-induced peripheral neuropathy (CIPN). CIPN is associated with psychological distress and a decrease of health-related quality of life (HRQoL), but little is known regarding bortezomib-related CIPN. This single center, cross-sectional study assessed the prevalence and severity of sensory/motor CIPN, neuropathic pain and ongoing pain medications, anxiety, depression, and HRQoL, in multiple myeloma patients after the end of bortezomib treatment. Paper questionnaires were sent to patients to record the scores of sensory and motor CIPNs (QLQ-CIPN20), neuropathic pain (visual analogue scale and DN4 interview), anxiety and depression (HADS), the scores of HRQoL (QLQ-C30 and QLQ-MY20) and ongoing pain medications. Oncological data were recorded using chemotherapy prescription software and patient medical records. The prevalence of sensory CIPN was 26.9% (95% CI 16.7; 39.1) among the 67 patients analyzed and for a mean time of 2.9 ± 2.8 years since the last bortezomib administration. The proportion of sensory CIPN was higher among patients treated by intravenous and subcutaneous routes than intravenous or subcutaneous routes (*p* = 0.003). QLQ-CIPN20 motor scores were higher for patients with a sensory CIPN than those without (*p* < 0.001) and were correlated with the duration of treatment and the cumulative dose of bortezomib (coefficient: 0.31 and 0.24, *p* = 0.01 and 0.0475, respectively), but not sensory scores. Neuropathic pain was screened in 44.4% of patients with sensory CIPN and 66.7% of them had ongoing pain medications, but none were treated with duloxetine (recommended drug). Multivariable analysis revealed that thalidomide treatment (odds-ratio: 6.7, 95% CI 1.3; 35.5, *p* = 0.03) and both routes of bortezomib administration (odds-ratio: 13.4, 95% CI 1.3; 139.1, *p* = 0.03) were associated with sensory CIPN. Sensory and motor CIPNs were associated with anxiety, depression, and deterioration of HRQoL. Sensory CIPN was identified in a quarter of patients after bortezomib treatment and associated with psychological distress that was far from being treated optimally. There is a need to improve the management of patients with CIPN, which may include better training of oncologists regarding its diagnosis and pharmacological treatment.

## Introduction

Chemotherapy-induced peripheral neuropathy (CIPN) is a common adverse effect of neurotoxic anticancer drugs, such as platinum derivative drugs (cisplatin, oxaliplatin), spindle poisons (taxanes: paclitaxel, docetaxel; vinca alkaloids: vincristine; epothilones; eribulin), bortezomib and thalidomide (Kerckhove et al., 2017). CIPN is commonly described as a distal and symmetric polyneuropathy (stocking and glove distribution). Overall symptomatology includes paresthesia (tingling, numbness), dysesthesia (thermal, tactile allodynia and neuropathic pain). The incidence of CIPN is close to 38%, but it can vary considerably according to the anticancer drugs and regimen prescribed (Kerckhove et al., 2017). CIPN remains a problematic adverse effect associated with a decline of health-related quality of life (HRQoL), and no preventive and unequivocally effective curative treatment (except duloxetine) is available today (Hershman et al., 2014; Loprinzi et al., 2020). Consequently, oncologists must decrease or stop the neurotoxic anticancer regimen to limit the severity of CIPN (Dault et al., 2016), possibly having a negative impact on disease control and progression free survival (Chibaudel et al., 2009).

Among all these neurotoxic anticancer drugs, bortezomib is probably one of the least studied whereas it is used in the treatment of multiple myeloma, the second most common hematologic malignancy after lymphoma (Kazandjian, 2016). Multiple myeloma arises from an asymptomatic premalignant proliferation of monoclonal plasma cells derived from post–germinal-center B cells. In the Western world, the age-standardized incidence of multiple myeloma has been reported to be approximately 5 cases per 100,000. The median age of patients at diagnosis is approximately 70 years (Palumbo and Anderson, 2011), and the five-year relative survival ratio, between 2003–2013, was 0.41 (95% CI 0.40; 0.43) (Thorsteinsdottir et al., 2018). According to the Cochrane database of Systematic Reviews, the increased risk (odds-ratio) of peripheral neuropathy in patients treated with bortezomib was 3.71 (95% CI 2.92; 4.70, *p* < 0.00001) (Scott et al., 2016). Li et al. described a median incidence of 37.8% for all grades of sensory CIPN for bortezomib treated patients in phase III clinical trials (Li et al., 2019). CIPN symptoms commonly include paresthesia and numbness occurring in the extremities, and progressing proximally in a glove and stocking distribution. Small nerve fiber involvement is common, characterized by pain in the toes and soles of the feet. CIPN is often under recognized in multiple myeloma patients. This can be explained in part by the fact that up to 54% of treatment-naïve patients demonstrated either clinical signs of peripheral neuropathy or presented abnormal neurophysiological results at baseline (Richardson et al., 2009). Moreover, bortezomib is often administered in combination with other drugs, such as thalidomide, which are also neurotoxic and have demonstrated a different mechanism of neurotoxicity. Consequently, the interaction of bortezomib with other treatments may lead to CIPN resulting from multiple pathophysiological pathways (Li et al., 2019).

Bortezomib-related CIPN is associated with a significant economic burden. A cost analysis based on US administrative claim databases showed a significantly higher healthcare utilization and expenditure per patient per month by $1509 for multiple myeloma patients with peripheral neuropathy than controls, driven by higher hospitalization (peripheral neuropathy 77.4%, controls 67.2%; *p* < 0.001) and emergency department rates (peripheral neuropathy 67.8%, controls 58.4%; *p* < 0.001) and more outpatient hospital-based visits (peripheral neuropathy 13.5 ± 14.7, controls 11.5 ± 18.0; *p* < 0.001) (Song et al., 2019).

The most commonly used evaluation tool for CIPN in clinical trials is the National *Cancer* Institute's common terminology criteria Adverse Reactions (NCI-CTCAE) (Li et al., 2019), which is a clinician-reported outcome (CROs) that includes criteria and definitions for quantifying the severity of CIPN in both sensory and motor components, utilizing a 5-point scale [grade 1 (asymptomatic) to grade 5 (death)] (Molassiotis et al., 2019a). However, this scale only demonstrates moderate inter-observer agreement (Postma et al., 1998) and is limited by floor and ceiling effects with limited responsiveness to change (Griffith et al., 2010). It is also well established that CROs underreport symptoms experienced by patients and that patient-reported outcomes (PROs) showed a higher incidence and severity of treatment-related toxicities, including CIPN (Beutler et al., 2017). Among the PROs, the QLQ-CIPN20 from the European Organization for Research and Treatment of *Cancer* (EORTC) is a valuable tool for the assessment of CIPN (Postma et al., 2005; Le-Rademacher et al., 2017).

To our knowledge, only 4 studies have assessed bortezomib associated CIPN with the QLQ-CIPN20 questionnaire for a total of 102 multiple myeloma patients treated by bortezomib: two studies including 80 patients (Beijers et al., 2016, 2017), another with 20 patients, (Mendoza et al., 2020), and the last one with 2 patients (Lavoie Smith et al., 2017). Moreover, very little information is available regarding the time since the last bortezomib administration. Only the study of Beijers et al. provides the time since the last chemotherapy administration (median: 10 months and range: 0–158) (Beijers et al., 2017). Thus, the prevalence and severity of bortezomib associated CIPN after the end of treatment has been studied only partially, likewise for psychological distress and HRQoL.

The aim of this study was to assess the prevalence and severity of CIPN associated with bortezomib based chemotherapy in patients with multiple myeloma, after the end of bortezomib administration. In addition, neuropathic pain, the use of pain medications, anxiety, depression, and HRQoL were assessed.

## Materials and Methods

### Study Design

This single center, observational and cross-sectional study was designed to assess CIPN in patients who were treated by bortezomib-based chemotherapy for multiple myeloma. The primary objective was the assessment of prevalence and severity of sensory CIPN. The secondary objectives were the severity of motor CIPN, the prevalence of neuropathic pain, the use of pain medications, the prevalence of anxiety and depression, and the HRQoL. Patients were assessed once and no longitudinal assessment was performed.

The study was designed according to the STROBE (Strengthening the Reporting of Observational Studies in Epidemiology) guidelines (von Elm et al., 2007). The study protocol was registered on the ClinicalTrials.gov website NCT03344328. The study was anonymous and approved by a local ethics committee (Comité de Protection des Personnes sud-est 5, IRB: 6705, No. 2017-A00651–52, april 25, 2017). The participants’ consent was obtained orally by contacting each eligible patient by phone.

The study design, the recruitment of patients and the data analysis were performed by the authors M.S., N.K. and D.B., none of whom were involved in the management of the patients included.

### Setting

This single center study was conducted in the rheumatology and clinical hematology departments of the University Hospital of Clermont-Ferrand (CHU Clermont-Ferrand). The inclusion of patients and data collection were carried out from March 13, 2019 until April 02, 2019.

### Participants

Inclusion criteria were patients having been treated by bortezomib-based chemotherapy for multiple myeloma. Exclusion criteria were patients <18 years, and neurological disease (stroke, Parkinson disease, Alzheimer disease, fibromyalgia).

Eligible patients were identified from the database of the chemotherapy prescription software (CHIMIO^®^, Computer Engineering, France) of the University Hospital of Clermont-Ferrand. A specific algorithm was prepared for the systematic extraction of patient data. Thereafter, this first selection of patients was checked by the oncologists in charge of patients, to select patients according to inclusion and exclusion criteria. Patients were phoned to inquire whether they would participate in the study. After patient acceptance, a paper questionnaire and a stamped envelope for the response were sent to the patient. Patients returned their questionnaires to the University Hospital of Clermont-Ferrand, where their responses were recorded and analyzed.

### Variables

The primary endpoints were the sensory score of the QLQ-CIPN20 rated from 0 (least) to 100 (worst) (Postma et al., 2005) (for scoring see: https://www.eortc.org/app/uploads/sites/2/2018/02/SCmanual.pdf), used as a quantitative variable, and the sensory CIPN defined as a sensory QLQ-CIPN20 score of ≥30/100 (Alberti et al., 2014; Selvy et al., 2020b), used as a qualitative variable. The secondary endpoints were the motor and vegetative scores of the QLQ-CIPN20 rated from 0 (least) to 100 (worst) (Postma et al., 2005). Present pain was reported by the patient (yes/no). Screening for pain was assessed with a visual analogue scale (VAS, 0 = no pain and 10 = maximum imaginable pain), and defined for a threshold ≥4/10. Among patients with a positive screening for pain (VAS score ≥4/10), neuropathic pain was assessed with DN4 interview (French abbreviation: Douleur Neuropathique 4, for neuropathic pain 4) questionnaire and detected for a DN4 interview score ≥3/7 (Bouhassira et al., 2005). The history of neuropathy and neuropathic pain before the diagnosis of multiple myeloma was also recorded. Ongoing pain medications in the past month were recorded, based on an established list of pain medications. Anxiety and depression were assessed with the Hospital Anxiety and Depression Scale (HADS) at the time of the answer, considering the following thresholds, normal (total score ≤7), borderline or suggestive of possible anxiety/depression (total score of 8–10) and indicative of anxiety/depression (total score ≥11) (Zigmond and Snaith, 1983). The HRQoL was assessed with the QLQ-C30 and QLQ-MY20 questionnaire (EORTC) (Aaronson et al., 1993; Cocks et al., 2007). The scoring of QLQ-C30 and QLQ-MY20 was done according to EORTC recommendations. The QLQ-C30 was divided into 3 subscales with a Global health status (0 worst to 100 best), the functional scales (0 worst to 100 best for physical functioning, role functioning, emotional functioning, cognitive functioning and social functioning) and the symptom scales (0 least to 100 worst for fatigue, nausea and vomiting, pain, dyspnea, insomnia, appetite loss, constipation, diarrhea and financial difficulties). The QLQ-MY20 was divided into 2 subscales with symptom scales (0 least to 100 worst for disease symptoms and side effects of treatment) and functional scales (0 worst to 100 best for body image and future perspective).

The oncological characteristics were recorded such as cumulative dose (mg/m^2^), route of administration (intravenous, subcutaneous), the date of the last bortezomib administration, the duration of bortezomib treatment, the administration of thalidomide, the date of the last thalidomide treatment, and the hematopoietic stem cell transplantation.

The socio-demographic characteristics of patients were also recorded such as gender, age, daily use of cigarettes, occasional alcohol use and hazardous alcohol use (>10 alcohol units per week) (France, 2017). Tobacco use has been associated to the severity of oxaliplatin-related CIPN in a previous study of our group (Selvy et al., 2020b). Alcohol use is a debated risk factor of CIPN (Molassiotis et al., 2019a).

### Data Sources/Measurement

Data assessing CIPN, neuropathic pain, ongoing pain medications, anxiety, depression, and HRQoL were obtained from the completed questionnaire. Oncological data and patient characteristics were obtained from the software of chemotherapy prescription and patient medical records. All the data were recorded and managed using REDCap™ electronic data capture tools hosted at the University Hospital of Clermont-Ferrand (Harris et al., 2009).

### Statistical Methods

The sample size was determined to ensure that the confidence interval (CI) of the sensory score of the QLQ-CIPN20 had an accuracy of around 5 points for a standard-deviation at 20. The calculation showed that at least 65 patients were necessary to ensure a two-sided type I error of 5%.

The internal consistency of the QLQ-CIPN20 sensory scale was assessed and determined using Cronbach’s *α* coefficient, with a minimum accepted value of 0.70. Then, the categorical data were presented using number of patients, percentage, and appropriate 95% CI. Continuous data were expressed as mean and standard-deviation. The normality of the data was assessed using the Shapiro–Wilk test. Continuous data were compared between independent groups (such as no sensory CIPN vs. sensory CIPN) using the Student’s t-test or the Mann–Whitney *U* test when the assumptions of the *t*-test were not met. The homoscedasticity of the data was assessed using the Fisher–Snedecor test. The results were expressed using Hedge’s effect-size (ES) and 95% CI, and were interpreted according to the rules of thumb reported by Cohen (Cohen, 1988), who defined the ES bounds as small (ES = 0.2), medium (ES = 0.5), and large (ES = 0.8). Categorical data were compared between groups (independent proportions) using the chi-squared test or Fisher’s exact test, and McNemar test for paired proportions. To analyze the relationships between continuous parameters, Pearson and Spearman correlation coefficients were estimated according to the statistical distribution of variables and by applying Sidak’s type I error correction, and interpreted as: <0.2 negligible, 0.2–0.4 weak, 0.4–0.7 moderate, >0.7 strong (Altman, 1999). To determine factors associated with the sensory CIPN (dependent variable), multivariable analysis (i.e. generalized linear logistic regression) was performed, including patients’ characteristics (gender, age, tobacco and alcohol consumptions) and characteristics of chemotherapy (time since last bortezomib administration, cumulative dose of bortezomib, and thalidomide treatment). Particular attention was paid to the study of multicollinearity and to the interactions between covariates: 1) studying the relationships between the covariables, and 2) evaluating the impact of adding or deleting variables on a multivariable model. The results are expressed as odds-ratios and 95% CI, and forest plots were used to present the results. Statistical analyses were performed using Stata 15 (StataCorp, College Station, US). All the tests were two-sided, with a type I error set at 5%. In accordance with the literature (Rothman, 1990; Bender and Lange, 2001; Feise, 2002), we reported all individual *p*-values without systematically applying any mathematical correction to the aforementioned tests comparing groups. Specific attention was given to the magnitude of differences (i.e., ES) and clinical relevance.

## Results

### Characteristics of Patients

**FIGURE 1:**
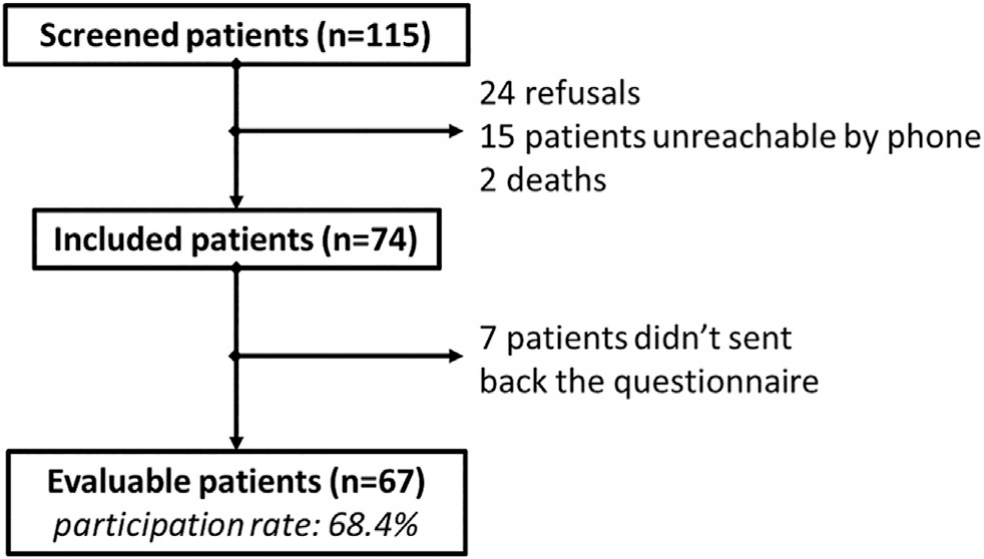
Flowchart.

One hundred and fifteen patients were screened by oncologists for inclusion in the study. Among them, 74 patients accepted to participate to the study and 67 sent back a filled questionnaire (response rate: 90.5%) (Figure 1). The characteristics of these 67 included patients are presented in Table 1.

**TABLE 1:** Characteristics of the analyzed patients (*N* = 67).

Items	Total *N* = 67	No sensory CIPN *N* = 49 (73.1)	Sensory CIPN *N* = 18 (26.9)	*p* Value	Effect size (95% CI)^#^
Female *n* (%)	35 (52.2)	27 (55.1)	8 (44.4)	0.44	−0.11 [−0.38; 0.16]
Age (years)	66.7 ± 10.4	66.2 ± 10.7	68.3 ± 9.7	0.44	0.20 [−0.33; 0.74]
Tobacco *n* (%)	3 (4.6)	2 (4.2)	1 (5.6)	0.81	0.01 [−0.11; 0.13]
Alcohol *n* (%)	32 (47.8)	25 (51.0)	7 (38.9)	0.38	−0.12 [−0.39; 0.14]
Hazardous alcohol use	4 (9.1)	3 (9.1)	1 (9.1)	1	0 [−0.20; 0.20]
History of neuropathic pain	40 (59.7)	29 (59.2)	11 (61.1)	0.89	−0.02 [−0.28; 0.24]
Bortezomib treatment	67 (100)				
Cumulative dose (mg/m²)	68.8 ± 41.9	61.8 ± 35.3	88.0 ± 52.8	0.12	0.64 [0.09; 1.18]
Duration of treatment (months)	15.5 ± 21.8	11.8 ± 18.0	25.5 ± 28.0	0.13	0.64 [0.10; 1.19]
Time since last administration (years)	2.9 ± 2.8	2.6 ± 2.7	3.7 ± 2.9	0.08	0.41 [−0.13; 0.94]
Subcutaneous route	50 (74.6)	41 (83.7)	9 (50.0)		
Intravenous route	9 (13.4)	6 (12.2)	3 (16.7)	0.003	0.04 [−0.15; 0.24]^##^
Both routes	8 (11.9)	2 (4.1)	6 (33.3)		0.29 [0.07; 0.52]^##^
Thalidomide treatment	35 (53.8)	23 (48.9)	12 (66.7)	0.20	0.18 [−0.8; 0.44]
Duration of treatment (months)	6.1 ± 3.2	6.5 ± 3.1	5.5 ± 3.4	0.39	−0.32 [−1.04; 0.40]
Time since last administration (years)	3.9 ± 2.6	3.2 ± 2.0	5.4 ± 3.1	0.0495	0.87 [0.12; 1.61]
Hematopoietic stem cell transplantation					
One transplantation	41 (62.1)	29 (60.4)	12 (66.7)	0.78	0.06 [−0.20; 0.32]
Two transplantations	13 (31.7)	9 (31.0)	4 (33.3)	1	0.02 [−0.29; 0.34]
QLQ-CIPN20 scores					
Sensory	18.9 ± 20.3	9.0 ± 10.5	45.7 ± 15.5	<0.001	3.02 [2.27; 3.76]
Motor	17.8 ± 20.7	10.9 ± 14.2	36.7 ± 23.9	<0.001	1.47 [0.88; 2.06]]
HADS scores					
Anxiety	5.6 ± 3.7	4.7 ± 3.7	7.9 ± 2.7	<0.001	0.90 [0.35; 1.46]
Depression	5.6 ± 4.4	4.5 ± 4.0	8.7 ± 3.7	<0.001	1.06 [0.49; 1.62]

Categorical variables are expressed as percentages (number). Continuous variables are expressed as mean ± standard deviation.

Standardized mean difference for continuous variables and absolute difference for categorical variables, and 95% confidence interval [95%CI].

Comparison vs. reference (subcutaneous route). Statistical analyzes were performed using student or Mann–Whitney tests for continuous variables and Chi-squared or Fisher’s exact tests for categorical variables.

### Sensory CIPN

The 20 items of the QLQ-CIPN20 indicated an excellent level of internal consistency (Cronbach *α* = 0.92), the 9 items of the sensory scale a good level (Cronbach *α* = 0.82), the 8 items of the motor scale indicated a good level (Cronbach *α* = 0.86) and the 3 items of the vegetative scale indicated a poor level (Cronbach *α* = 0.65). Thereafter, the vegetative scale of the QLQ-CIPN20 was not used for the analysis.

**FIGURE 2:**
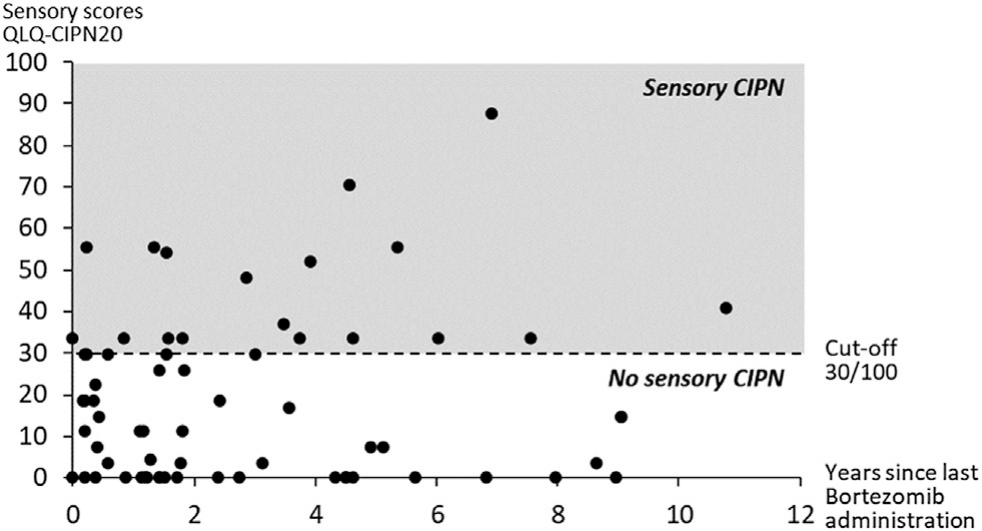
Distribution of the sensory scores of the QLQ-CIPN20 for each patient and over years after the end of bortezomib administration.

Among the patients analyzed, 26.9% 18) (95% CI 16.7; 39.1) had a sensory CIPN (sensory QLQ-CIPN20 score ≥30/100). The distribution of the sensory scores of the QLQ-CIPN20 over the years after the end of chemotherapy is presented in Figure 2. Sensory scores were not different between males and females (20.1 ± 19.9 vs. 17.8 ± 20.8, *p* = 0.51) and not correlated with the age of patients (Table 2).

**TABLE 2:** Correlations between quantitative variables and the sensory scores of the QLQ-CIPN20 and the motor scores of the QLQ-CIPN20.

QLQ-CIPN20 sensory scores	Spearman coefficient	*p* values
**Age** (years)	0.11	>0.05
**Bortezomib treatment**		
Cumulative dose (mg/m²)	0.19	0.12
Duration of treatment (months)	0.19	0.12
Time since last administration (years)	0.04	0.72
**Thalidomide treatment**		
Duration of treatment (months)	0.13	0.47
Time since last administration (years)	0.26	0.16
**QLQ-CIPN20 motor scores**		
**Age** (years)	0.21	>0.05
**Bortezomib treatment**		
Cumulative dose (mg/m²)	0.24	0.0475
Duration of treatment (months)	0.31	0.0099
Time since last administration (years)	−0.16	0.18
**Thalidomide treatment**		
Duration of treatment (months)	−0.06	0.77
Time since last administration (years)	0.23	0.21

Characteristics of bortezomib treatments (cumulative dose, duration, and time since last administration) were not different between patients with or without a sensory CIPN (Table 1). Likewise, sensory scores were not correlated with the cumulative bortezomib dose, with the duration of bortezomib treatment, or with the time since last bortezomib administration (Table 2 and Figure 2). The proportions of sensory CIPN were significantly different according to the route of bortezomib administration (*p* = 0.003) (Table 1). Post hoc analysis revealed a higher proportion of sensory CIPN in patients with both routes of bortezomib administration (intravenous + subcutaneous) compared to the subcutaneous route only (*p* < 0.05). Interestingly, the proportions of sensory CIPN were not different between intravenous and subcutaneous routes. Similarly, the sensory scores of the QLQ-CIN20 were different according to the route of bortezomib administration (intravenous vs. subcutaneous vs. both routes: 21.7 ± 28.9 vs. 15.8 ± 17.7 vs. 35.2 ± 18.8, *p* = 0.02).

**FIGURE 3:**
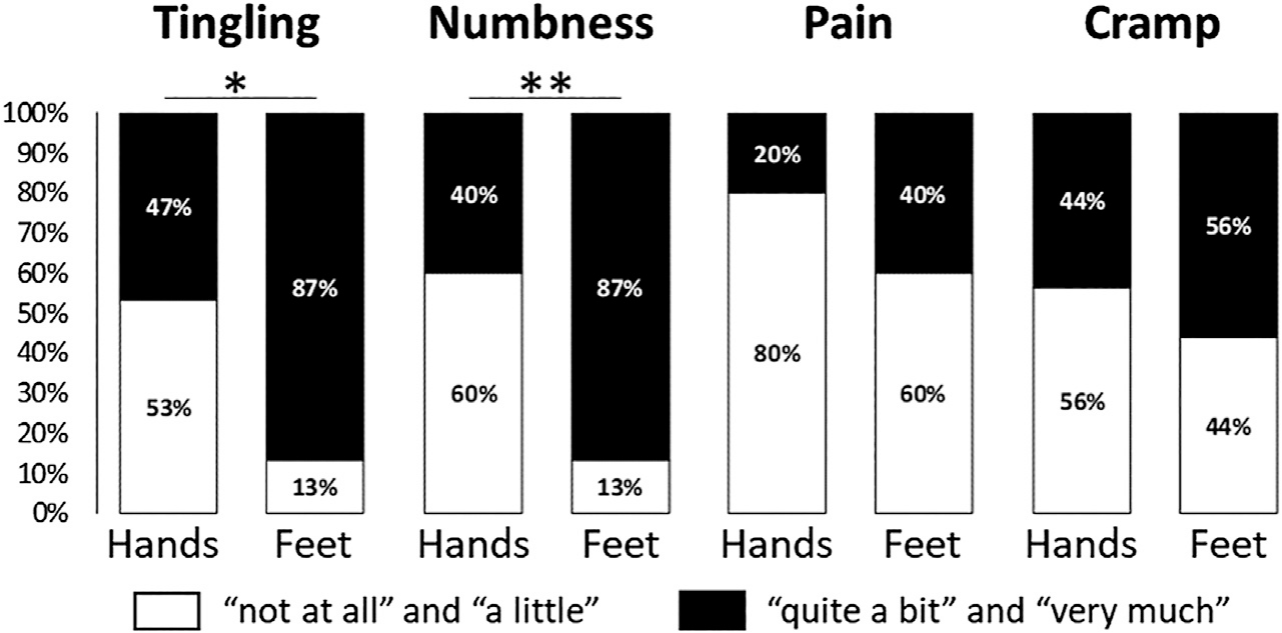
Severity proportions of the QLQ-CIPN20 items assessing tingling, numbness, pain and cramp in hands and feet, among patients with a sensory CIPN. The response categories were recoded to yield a dichotomous outcome per item (white: “not at all” and “a little” vs. black: “quite a bit” and “very much”). *p < 0.05, **p < 0.01. Statistical analysis was performed using McNemar test for paired proportions.

Proportions of patients with a sensory CIPN and sensory scores (20.0 ± 22.4 vs. 17.5 ± 18.3, *p* = 0.95) were not different between patients treated or not with thalidomide (Table 1). The durations of thalidomide treatment were not different between patients with or without a sensory CIPN (Table 1), and were not correlated with the sensory scores (Table 2). Times since last thalidomide administration were longer for patients with a sensory CIPN than patients without a sensory CIPN (Table 1), but were not correlated with the sensory scores (Table 2). Moreover, proportions of patients with a sensory CIPN and the sensory scores were not different between patients having received a hematopoietic stem cell transplantation or not (for proportion see Table 1, and 17.8 ± 21.6 vs. 20.2 ± 18.5, *p* = 0.42, respectively). Finally, among patients with a sensory CIPN, tingling and numbness were proportionally higher in feet than in hands (*p* < 0.05) (Figure 3).

**FIGURE 4:**
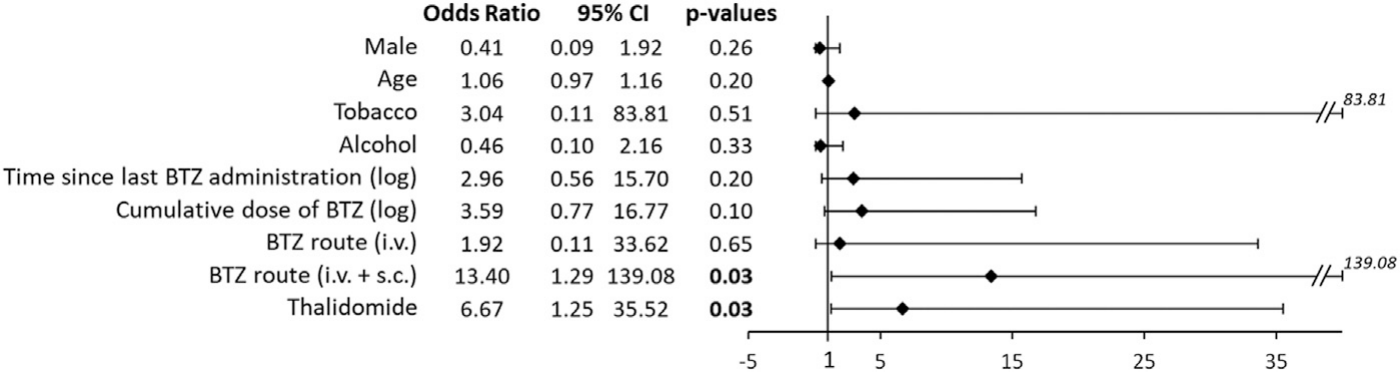
Forrest plot of the regression coefficients comparing sensory CIPN with patient characteristics and treatments. Multivariable analyses were performed, including patient characteristics: gender (male vs. female), age, tobacco, and alcohol; and chemotherapy characteristics: time since last bortezomib (BTZ) administration (logarithmic transformation), BTZ cumulative dose (logarithmic transformation), route of BTZ administration (intravenous (i.v.) vs. subcutaneous (s.c.) and i. v. + *s*. c. vs. s. c.), and thalidomide administration. Statistical analysis was performed using multivariable logistic regression.

In parallel, a multivariable analysis of the sensory CIPN was performed on associated factors (male, age, tobacco, alcohol, time since last bortezomib administration, bortezomib routes, and thalidomide treatment). Bortezomib administration via intravenous + subcutaneous routes was associated with a higher proportion of patients with a sensory CIPN, compared to other routes (intravenous route only or subcutaneous route only). Thalidomide treatment was associated with a higher proportion of patients with a sensory CIPN compared to no thalidomide treatment (Figure 4).

### Motor CIPN

Motor scores of the QLQ-CIPN20 were strongly correlated with sensory scores (Spearman coefficients: 0.74, *p* < 0.05), were higher for patients with a sensory CIPN than patients without a CIPN (36.7 ± 23.9 vs. 10.9 ± 14.2, *p* < 0.001), were not different between males and females (15.1 ± 19.7 vs. 20.4 ± 21.5, *p* = 0.3), and were not correlated with age of patients (Table 2). Motor scores were weakly correlated with the duration and the cumulative dose of bortezomib treatment, but not with the time since last bortezomib administration (Table 2). Motor scores were not different between patients having been treated by thalidomide or not (18.7 ± 20.4 vs. 17.0 ± 21.9, *p* = 0.65), and were not correlated with the time since last thalidomide administration, or the duration of thalidomide treatment (Table 2). Lastly, motor scores were not different between patient having received a hematopoietic stem cell transplantation or not (15.2 ± 20.0 vs. 21.9 ± 21.8, *p* = 0.11).

### Neuropathic Pain and Pain Medications

History of neuropathy and neuropathic pain was similar between patients with or without sensory CIPN. However, this data must be interpreted cautiously because patients tended to mix past neuropathy history, actual CIPN and multiple myeloma symptoms.

Among the patients analyzed, 59.7% (40) declared having present pain (yes/no), 25.4% 17) were screened positively for pain (pain VAS ≥4/10), and 14.9% (10) for neuropathic pain (pain VAS ≥4/10 and DN4 interview ≥3/7). Among patients with a sensory CIPN, 50.0% 9) were screened positively for pain, and 44.4% 8) for neuropathic pain; the latter proportion was higher than for patients without a sensory CIPN (4.1%, *p* < 0.001). Sensory and motor scores were higher for patients with a neuropathic pain than patients without (Sensory: 46.5 ± 21.7 vs. 14.1 ± 15.7, *p* < 0.001; Motor: 38.8 ± 19.8 vs. 14.2 ± 18.7, *p* < 0.001).

Finally, 52.0% of all the patients analyzed and 66.7% of patients with a sensory CIPN declared they took pain medications (Table 3). Most of the patients both in the sensory CIPN and neuropathic pain groups, declared to take paracetamol (75.0 and 62.5%, respectively), which was also similar to patients without sensory CIPN or neuropathic pain. No patient received duloxetine, one patient with a sensory CIPN received gabapentin and another one pregabalin. It is noteworthy that two patients without sensory CIPN took pregabalin (Table 3).

**TABLE 3:** Ongoing analgesic treatments among all the patients included, patients without or with a sensory CIPN and patients without or with neuropathic pain. Categorical variables are expressed as numbers (%).

	Total (*N* = 67)	No sensory CIPN (*N* = 49)	Sensory CIPN (*N* = 18)	No neuropathic pain (*N* = 57)	Neuropathic pain (*N* = 10)
Analgesic treatment	35 (52.2)	23 (46.9)	12 (66.7)	27 (47.4)	8 (80.0)
Paracetamol	26 (74.3)	17 (73.9)	9 (75.0)	21 (77.8)	5 (62.5)
Aspirin	5 (14.3)	4 (17.4)	1 (8.3)	4 (14.8)	1 (12.5)
Morphine	4 (11.4)	3 (13.0)	1 (8.3)	3 (11.1)	1 (12.5)
Tramadol + paracetamol	3 (8.6)	2 (8.7)	1 (8.3)	3 (11.1)	0 (0)
Pregabalin	3 (8.6)	2 (8.7)	1 (8.3)	3 (11.1)	0 (0)
Ibuprofen	2 (5.7)	2 (8.7)	0 (0)	2 (7.4)	0 (0)
Codeine + paracetamol	2 (5.7)	0 (0)	2 (16.7)	0 (0)	2 (25.0)
Tramadol	2 (5.7)	2 (8.7)	0 (0)	2 (7.4)	0 (0)
Opium + paracetamol	2 (5.7)	1 (4.4)	1 (8.3)	1 (3.7)	1 (12.5)
Paracetamol + opium + caffeine	1 (2.9)	1 (4.4)	0 (0)	0 (0)	1 (12.5)
Gabapentin	1 (2.9)	0 (0)	1 (8.3)	0 (0)	1 (12.5)
Amitriptyline	1 (2.9)	0 (0)	1 (8.3)	1 (3.7)	0 (0)
Codeine	0 (0)	0 (0)	0 (0)	0 (0)	0 (0)
Duloxetine	0 (0)	0 (0)	0 (0)	0 (0)	0 (0)
Dihydrocodeine	0 (0)	0 (0)	0 (0)	0 (0)	0 (0)
Imipramine	0 (0)	0 (0)	0 (0)	0 (0)	0 (0)

### Impact of CIPN on Anxiety, Depression and Quality of Life

**FIGURE 5:**
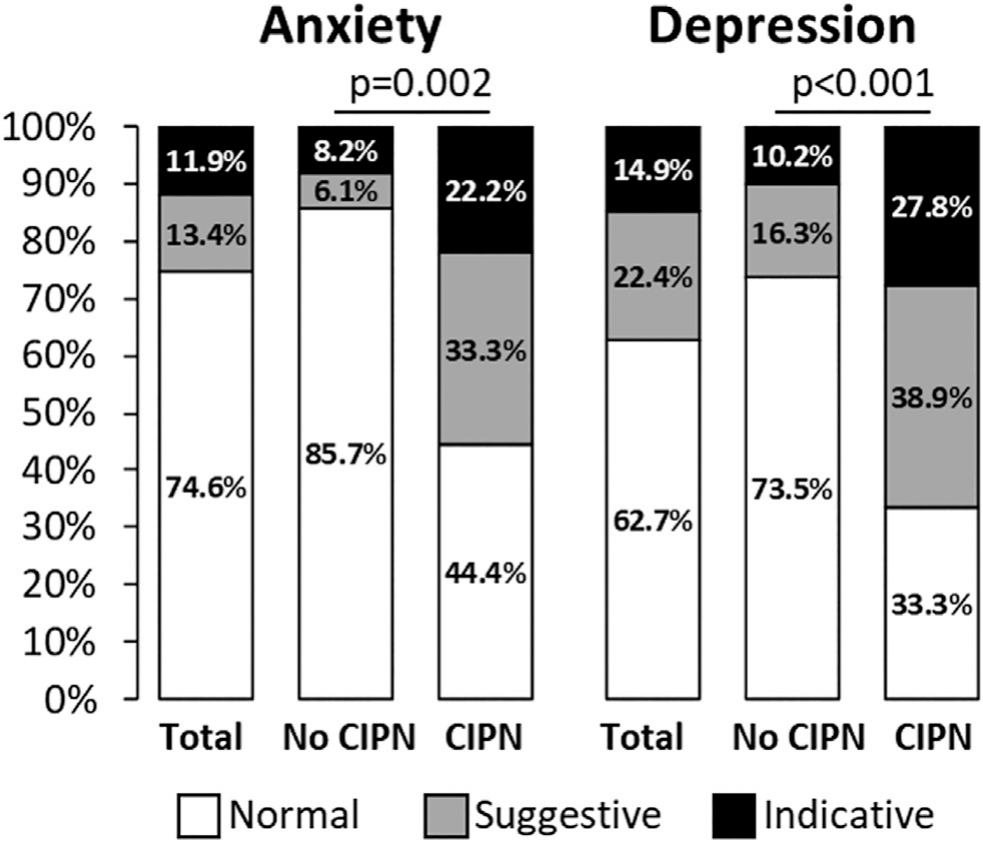
Proportion of anxiety and depression according to sensory CIPN. The results are expressed as percentages. Normal scores of HADS were ≤7, suggestive 8–10 and indicative ≥11 for anxiety or depression. Statistical analysis was performed using Fisher’s exact test.

Proportions of anxiety and depression were higher in patients with sensory CIPN than those without (Figure 5). Sensory scores were higher among patients with anxiety or depression disorders (normal vs. suggestive vs. indicative scores of anxiety: 13.2 ± 15.4 vs. 34.2 ± 22.2 vs. 37.1 ± 27.3, *p* < 0.001; normal vs. suggestive vs. indicative scores of depression: 11.4 ± 15.9 vs. 34.5 ± 22.0 vs. 27.1 ± 18.8, *p* < 0.001). The same results were observed for motor scores (normal vs. suggestive vs. indicative scores of anxiety: 12.0 ± 16.2 vs. 34.1 ± 23.3 vs. 35.9 ± 24.8, *p* < 0.001; normal vs. suggestive vs. indicative scores of depression: 9.9 ± 15.4 vs. 26.5 ± 20.2 vs. 38.2 ± 23.3, *p* < 0.001).

The scores of sensory CIPN were moderately correlated with the scores of quality of life of the QLQ-C30 questionnaire for several dimensions (global health status, physical functioning, role functioning, emotional functioning, cognitive functioning, social functioning, pain and insomnia) (Table 4). Moreover, the scores of these dimensions were significantly different between patients with a sensory CIPN and those without (Table 4). The scores of sensory CIPN were moderately to strongly correlated with the scores of all the dimensions of the QLQ-MY20 (disease symptoms, side effects of treatment, body image and future perspective) and the scores of these dimensions were different between patients with a sensory CIPN and those without (Table 4).

**TABLE 4:** Scores of quality of life (QLQ-C30 and QLQ-MY20) according to sensory CIPN, and correlation with sensory and motor scores of the QLQ-CIPN20.

	**All the patients**	**No sensory CIPN**	**Sensory CIPN**	**Effect size [CI 95%]**	**Correlations sensory scores**	**Correlations motor scores**
QLQ-C30						
Global health status	63.5 ± 24.1	68.1 ± 22.7	51.9 ± 24.3*	−0.69 [−1.24; −0.14]	−0.52*	−0.60*
Physical functioning	74.5 ± 23.0	79.5 ± 22.5	60.0 ± 18.3***	−0.90 [−1.46; −0.33]	−0.66*	−0.65*
Role functioning	75.8 ± 29.7	83.0 ± 25.8	54.9 ± 31.0**	−1.02 [−1.59; −0.44]	−0.56*	−0.59*
Emotional functioning	74.8 ± 26.2	77.8 ± 26.1	67.0 ± 25.5*	−0.42 [−0.95; 0.13]	−0.47*	−0.58*
Cognitive functioning	83.3 ± 22.5	87.5 ± 19.3	72.2 ± 26.8*	−0.70 [−1.25; −0.15]	−0.48*	−0.59*
Social functioning	71.3 ± 27.7	77.3 ± 25.6	55.6 ± 27.4**	−0.82 [−1.37; −0.26]	−0.59*	−0.70*
Fatigue	36.5 ± 31.4	30.2 ± 30.1	53.7 ± 28.8**	0.78 [0.23; 1.33]	0.51	0.65*
Nausea and vomiting	7.1 ± 17.8	6.5 ± 14.8	8.8 ± 25.1	0.13 [−0.42; 0.67]	0.11	0.30*
Pain	25.0 ± 27.6	18.1 ± 24.0	43.5 ± 28.7^c^	0.99 [0.43; 1.55]	0.54*	0.58*
Dyspnea	28.3 ± 29.4	27.2 ± 26.9	31.4 ± 36.3	0.14 [−0.41; 0.68]	0.19	0.29*
Insomnia	35.4 ± 35.0	27.9 ± 32.9	56.9 ± 32.8**	0.87 [0.30; 1.43]	0.49*	0.61*
Appetite loss	18.7 ± 31.6	16.3 ± 29.7	25.5 ± 36.4	0.29 [−0.26; 0.83]	0.19	0.40*
Constipation	27.8 ± 33.9	24.5 ± 31.7	37.2 ± 38.9	0.37 [−0.18; 0.92]	0.27*	0.37*
Diarrhea	16.4 ± 29.2	15.0 ± 28.1	20.4 ± 32.6	0.18 [−0.35; 0.72]	0.13	0.36*
Financial difficulties	8.6 ± 21.3	5.6 ± 17.3	16.7 ± 28.6	0.52 [−0.02; 1.07]	0.29*	0.35*
QLQ-MY20						
Disease symptoms	20.8 ± 20.5	15.8 ± 16.2	34.6 ± 25.0**	0.98 [0.42; 1.54]	0.55*	0.58*
Side effects of treatment	22.4 ± 21.1	16.3 ± 17.4	38.9 ± 21.8***	1.19 [0.62; 1.76]	0.74*	0.76*
Body image	75.4 ± 34.0	83.0 ± 30.2	55.6 ± 36.1**	−0.85 [−1.40; −0.29]	−0.44*	−0.62*
Future perspective	61.2 ± 31.7	67.8 ± 29.2	43.8 ± 32.0**	−0.79 [−1.34; −0.23]	−0.47*	−0.49*

The results present the Spearman coefficient (correlation) between the sensory and motor scores of the QLQ-CIPN20 and the scores of the QLQ-C30 and the scores of the QLQ-MY20. The mean (±standard deviation) scores of the QLQ-C30 dimensions and the QLQ-MY20 dimensions are presented for all the patients, the patients with no sensory CIPN and the patients with a sensory CIPN. Effect size (Hedge coefficient) and 95% interval confidence of this comparison are presented.

* *p* < 0.05.

** *p* < 0.01.

*** *p* < 0.001.

No sensory CIPN vs. sensory CIPN. Statistical analyzes were performed using student or Mann–Whitney tests for the comparisons between sensory CIPN (yes vs no), and Spearman correlation coefficient to the study of relationship between QLQ-C30, QLQ-MY20 and sensory/motor scores of the QLQ-CIPN20.

The scores of motor CIPN were moderately correlated with the scores of each dimension of the QLQ-C30 questionnaire (except: nausea and vomiting, dyspnea, constipation, diarrhea, and financial difficulties, which had weak correlations) (Table 4). The scores of motor CIPN were moderately to strongly correlated with the scores of all the dimensions of the QLQ-MY20 (Table 4).

## Discussion

A preliminary finding was that the analysis of the QLQ-CIPN20 questionnaire revealed good internal validity of the sensory and motor scales. In contrast, the results for the vegetative scale were questionable with a poor level of internal consistency, which has already been reported by other authors (Smith et al., 2019).

Among the 67 patients analyzed having completed bortezomib treatment for multiple myeloma, 26.9% had a sensory CIPN after a median of 1.8 years of bortezomib treatment (min: 0 and max: 10.8 years). In a recent meta-analysis of phase III randomized controlled trials involving bortezomib in any treatment arm for the treatment of multiple myeloma, the overall incidence of sensory peripheral neuropathy ranged from 8.4 to 80.5% (median = 37.8%) for all grades, and from 1 to 33.2% (median = 8%) for grade 3–4 (Li et al., 2019). In another study assessing the long-term outcomes (median follow-up time 24 months) of 128 bortezomib treated Chinese patients, the overall incidence of peripheral neuropathy was 48.4% for all grades, and 17.1% for grade 3–4 (Xia et al., 2016). Another study, focusing on CIPN in multiple myeloma patients, reported 65% of grade 2–3 CIPN assessed with the Indication for Common Toxicity Criteria Grading of Peripheral Neuropathy Questionnaire, which is a PRO (Beijers et al., 2016).

The sensory scores of the QLQ-CIPN20 and proportion of patients with a sensory CIPN were not related to the time since last bortezomib administration. These results suggested that this CIPN does not completely regress over time, unlike in other studies (Richardson et al., 2006). Our small number of patients may limit this interpretation. Nevertheless, this difference could be explained by the fact that screening and diagnosing CIPN are highly dependent on the tools and methods used. PROs identified a higher incidence and severity of treatment-related toxicities including CIPN than CROs (Beutler et al., 2017). Moreover, another study of our group with the same design and assessing oxaliplatin-related CIPN in a cohort of 406 patients showed a decrease of the QLQ-CIPN20 sensory scores (*p* = 0.048) but not of the prevalence of the sensory CIPN, over 5 years after the end of chemotherapy (Selvy et al., 2020b).

The characteristics of bortezomib treatments (cumulative dose and duration of treatment) did not influence neuropathy, as described previously (Beijers et al., 2017; Li et al., 2019). Our study revealed a higher proportion of sensory CIPN in patients with both routes of bortezomib administration (intravenous + subcutaneous) compared to the subcutaneous route, but not between intravenous and subcutaneous routes. Noteworthy, ES were weak to negligible between patients with or withour sensory CIPN. In the same way, Minarik et al. did not find any improvement in the incidence of peripheral neuropathy according to the subcutaneous route (Minarik et al., 2015). However, the subcutaneous route has also been described as more neuroprotective than the intravenous route (Peng et al., 2015). It should be noted that in the present study, the number of patients having been treated only by intravenous route was probably too small to show a statistical significance in comparison to other routes of administration.

In the multivariable analysis, thalidomide treatment was related to the severity of the sensory CIPN. This result has already been described in the literature (Li et al., 2019) since thalidomide is also a neurotoxic anticancer drug (Velasco et al., 2019).

Sensory CIPN was associated with a higher severity of motor CIPN, with a large ES. Besides being toxic to motor nerves, bortezomib can induce a myopathy with symmetrical and proximal lower limb muscle weakness, and without alteration of serum creatine kinase. Bortezomib-induced myopathy completely resolves after treatment discontinuation (Guglielmi et al., 2017). Bortezomib-induced myopathy may result from mitochondrial toxicity inducing or associating lipid accumulation within muscle fibers (Guglielmi et al., 2017).

The severity of the sensory CIPN was associated with neuropathic pain. Nearly half (44.4%) of the patients with a sensory CIPN had neuropathic pain. These results are very close to those of Lakshman et al. and Corso et al., who found a prevalence between 40 and 50% according to the NCI CTCAE (Corso et al., 2010; Lakshman et al., 2017). Comparatively, neuropathic pain seems to be lower in other types of CIPN, oxaliplatin-induced peripheral neuropathy (36.5% (Selvy et al., 2020b) and 20% (de Carvalho Barbosa et al., 2014)) and paclitaxel-induced peripheral neuropathy (22.5–50% (Golan-Vered and Pud, 2013)). Multiple myeloma is by itself a painful condition, because of osteolytic bone lesions, with back localization in over three quarters of patients. Such lesions are one of the most common complications of multiple myeloma. Myeloma bone disease affects up to 90% of patients complaining of bone pain (Coluzzi et al., 2019). This may be a confounding factor in the assessment of neuropathic pain.

Due to the pain component of the CIPN and to multiple myeloma, the patients included received mainly conventional analgesic treatments. Interestingly, two patients without sensory CIPN declared to take pregabalin, which could be interpreted that pregabalin was effective to treat neuropathic symptoms in these two patients. Duloxetine, which remains the only treatment recommended by the American Society of Clinical Oncology (ASCO) for CIPN (Hershman et al., 2014; Loprinzi et al., 2020), was not used by oncologists to treat CIPN. There appears to be no clear or robust explanation capable of justifying this lack of management. The ASCO guidelines for the management of CIPN (Hershman et al., 2014; Loprinzi et al., 2020) may not have been correctly disseminated to French oncologists, a consideration also mentioned in another study of our group (Selvy et al., 2020b). A Japanese study demonstrated that the dissemination of the Japanese Clinical Guidelines for the Management of CIPN in 2017 (CIPN-GL2017), incorporating ASCO recommendations, increased the prescription rate of duloxetine by Japanese oncologists, for the management of CIPN (Hirayama et al., 2020). Moreover medications used for the management of peripheral neuropathic pain (antiepileptics and antidepressants) are associated with many adverse effects which are underestimated (Selvy et al., 2020a), and which may decrease patient adherence to treatment (Oladapo et al., 2012; Timmerman et al., 2016). Finally, the diagnosis of CIPN is still a concern, as there is no clear consensus on a robust and easy-to-use tool (Colvin, 2019), so perhaps difficulties in the diagnosis and treatment have led to under-diagnosing and under-treating these patients.

In this population of patients with multiple myeloma and treated with bortezomib, sensory and motor CIPNs were strongly associated with depression, anxiety, and a reduced HRQoL, with large ESs for most of the QLQ-C30 and QLQ-MY20 items. To our knowledge, no publication has explored the association of CIPN and anxiety. Only two publications presented results between CIPN and depression (Beijers et al., 2016; Azoulay et al., 2019). In a cohort of 289 multiple myeloma patients, bone pain and peripheral neuropathy were the two leading symptoms interfering with daily life (Zaleta et al., 2020). Our findings are also shared by other types of CIPNs (Simon et al., 2017; Bonhof et al., 2019; Soveri et al., 2019; Selvy et al., 2020b) and they confirmed Beijers' findings (Beijers et al., 2016) that CIPN significantly alters patients' quality of life over the long term. However, number of treatment lines, disease and treatment status were not recorded in the study, whereas these parameters could also influence HRQoL and psychological distress (Despiégel et al., 2019). Importantly, scores of the QLQ-C30 and HADS questionnaire in our study were very close to those of the study of Servadio et al. assessing HRQoL in 99 multiple myeloma survivors up to 11 years after diagnosis (Servadio et al., 2019).

### Limitations of the Study

The use of the QLQ-CIPN20 questionnaire to assess the prevalence of CIPN may be controversial. In the present study, based on a work by Alberti et al., we used QLQ-CIPN20 scoring to approximate the prevalence of sensory CIPN, considering a QLQ-CIPN20 threshold of ≥30/100 to approximate a grade ≥2 sensory CIPN (Alberti et al., 2014). Alberti et al. showed a close relation between QLQ-CIPN20 scores and NCI-CTCAE sensory grade (*p* < 0.001). QLQ-CIPN20 scores between 30 and 40 (median ≈ 35, interquartile range ≈ 26–50, and mean ≈ 39) were associated with an NCI-CTCAE sensory neuropathy grade 2 and QLQ-CIPN20 scores >40 (median ≈ 59, interquartile range ≈ 39–62, and mean ≈ 57) were associated with an NCI-CTCAE sensory neuropathy grade 3/4 (Alberti et al., 2014). The QLQ-CPIN20 scoring was able to discriminate NCI-CTCAE neuropathy grade 1 vs. grade 2 (*p* < 0.001), and grade 2 vs. grade 3/4 (*p* < 0.001). However, the QLQ-CIPN20 scoring was not able to discriminate neuropathy grade 0 vs. grade 1 (*p* = 0.53). Le-Rademacher et al. concluded that there are no QLQ-CIPN20 score ranges that correspond directly with NCI-CTCAE grading levels (Le-Rademacher et al., 2017). However, they also emphasized that the QLQ-CIPN20 provided detailed information, distinguished more subtle degrees of neuropathy, and that it was more responsive to change over time than the NCI-CTCAE (Le-Rademacher et al., 2017). Importantly, the sensory score of the QLQ-CIPN20 of the present study (18.9 ± 20.3) were very close to those of the studies of Mendoza et al. (17.2 ± 4.7) (Mendoza et al., 2020) and Beijers et al. (15.3 ± 16.7) (Beijers et al., 2017). In our study, the self-administered questionnaire was particularly useful to assess CIPN severity using a paper questionnaire sent to patients. Neuropathy history was initially recorded but data were excluded from the analysis because the patients tended to mix past neuropathy history and ongoing CIPN. However, neuropathy history would have been of interest, because it has already been described as a risk factor of CIPN (Molassiotis et al., 2019b). Selection bias may be present in the study, since the patients came from a single center. However, the patients were managed in two different medical departments of the university hospital of Clermont-Ferrand. It is also possible that neuropathic patients are over-represented because these patients have felt compelled to respond to the questionnaires. Information bias was probably present, since the patients’ answers were subjective and unsupported by clinical assessment (such as neurological examination, nerve conduction studies, quantitative sudomotor axon reflex test or skin biopsies). Moreover, PRO measures may overestimate CIPN prevalence as they include symptoms that may have pre-existed the chemotherapy (Molassiotis et al., 2019b). Although the oncological data came from the medical prescription software of the university hospital.

## Conclusion

Sensory CIPN was identified in a quarter of the patients after ending bortezomib treatment, underlining the high prevalence and persistence of this adverse effect. Interestingly, neuropathic pain was highly prevalent in patients with a sensory CIPN, unlike CIPN associated with other neurotoxic anticancer drugs. The bortezomib-related CIPN was associated with considerable psychological distress. Management of patients with a sensory CIPN was not adequate, a finding in agreement with other studies, and which highlights the global lack of management of CIPN in France. There is a need to improve CIPN management which could include better screening, treatment and follow-up of patients. Such strategy should also include a better training for oncologists, since French oncologists’ professional practices are not optimal (Selvy et al., 2021).

CIPN considerably decreases the HRQoL of cancer patients and there is a current lack of innovative strategies for both assessing and managing it.

## Data Availability

The raw data supporting the conclusions of this article will be made available by the authors, upon request.
